# The Influence of Pineapple Leaf Fiber Orientation and Volume Fraction on Methyl Methacrylate-Based Polymer Matrix for Prosthetic Socket Application

**DOI:** 10.3390/polym13193381

**Published:** 2021-09-30

**Authors:** Eric Worlawoe Gaba, Bernard O. Asimeng, Elsie Effah Kaufmann, E. Johan Foster, Elvis K. Tiburu

**Affiliations:** 1 Department of Biomedical Engineering, School of Engineering Sciences, University of Ghana, Accra P.O. Box LG 74, Ghana; ewgaba@st.ug.edu.gh (E.W.G.); boasimeng@ug.edu.gh (B.O.A.); eeffahkaufmann@ug.edu.gh (E.E.K.); 2 Department of Orthotics and Prosthetics, School of Allied Health Sciences, University of Health and Allied Sciences, Ho PMB 31, Ghana; 3 Department of Chemical and Biological Engineering, University of British Columbia, 2360 East Mall, Vancouver, BC V6T 1Z3, Canada; johan.foster@ubc.ca; 4 School of Optometry and Vision Science, Faculty of Medicine and Health, University of New South Wales, Sydney, NSW 2052, Australia

**Keywords:** prosthetic socket, pineapple leaf fiber, orientation, volume fraction, flexural properties

## Abstract

This work reports on the use of low-cost pineapple leaf fiber (PALF) as an alternative reinforcing material to the established, commonly used material for prosthetic socket fabrication which is carbon-fiber-reinforced composite (CFRC) due to the high strength and stiffness of carbon fiber. However, the low range of loads exerted on a typical prosthetic socket (PS) in practice suggests that the use of CFRC may not be appropriate because of the high material stiffness which can be detrimental to socket-limb load transfer. Additionally, the high cost of carbon fiber avails opportunities to look for an alternative material as a reinforcement for composite PS development. PALF/Methyl Methacrylate-based (MMA) composites with 0°, 45° and 90° fiber orientations were made with 5–50 *v*/*v* fiber volume fractions. The PALF/MMA composites were subjected to a three-point flexural test to determine the effect of fiber volume fraction and fiber orientation on the flexural properties of the composite. The results showed that 40% *v*/*v* PALF/MMA composite with 0° fiber orientation recorded the highest flexural strength (50 MPa) and stiffness (1692 MPa). Considering the average load range exerted on PS, the flexural performance of the novel composite characterized in this work could be suitable for socket-limb load transfer for PS fabrication.

## 1. Introduction

A lower-limb prosthesis is an artificial limb support mechanism designed to restore the lower-limb mobility of an amputee [1], with the most essential and amputee-specific part being the prosthetic socket (PS) [2]. The PS is the part of the prosthetic assemblage that accommodates and interfaces with the amputee’s residual limb. Biomechanically, the PS is responsible for load-bearing and load transfer to downstream prosthetic components such as the pylon and the prosthetic foot [3,4]. The product fabrication specifications for a PS require the socket to be: (a) strong enough to bear the load of the amputee, (b) stiff to resist bending and shear stresses, (c) flexible to absorb torque, (d) durable to resist fractures and stress in multiple planes, (e) lightweight to reduce the energy expenditure of the amputee and (f) cost-effective [2,3,5,6]. To achieve these multifaceted properties for a PS, the type of material selected for the fabrication is important. Thermoplastic and thermosetting plastics are the two materials used for PS fabrication [7]. However, thermosetting (composite) plastics are preferred because of the opportunity to incorporate multiple materials with desired properties whose combined effect satisfies the product fabrication specification for the PS.

Carbon fiber, fiberglass, stockinette (cotton derived material to improve socket flexibility), polyester and acrylic resin are the known materials used for PS fabrication [7,8]. These materials are categorized into two: matrix and reinforcing material [6]. The matrix consists of either polyester or acrylic resin that binds the reinforcing elements together to form a brittle-fiber ductile-matrix system [9]. The combined effect of the fiber-matrix system enhances load transfer from the proximal end of the socket to the distal end where it is attached to the pylon. While the fiberglass, cotton stockinette and matrix introduce an amount of flexibility into the PS to absorb torque, the carbon fiber introduces very high strength and stiffness into the PS to bear the weight exerted on the socket and to accommodate the flexural stresses transmitted through the socket wall during gait cycle [3,5]. Compared to other alternatives such as bio-fibers, carbon fiber and fiberglass materials are also expensive. This makes the overall cost of fabricating a high-quality PS expensive, especially for amputees in resource-constrained parts of the world [10,11]. These challenges have therefore created the need to explore low-cost, renewable, eco-friendly and sustainable sources for engineering bio-fibers. Among the family of bio-fibers, plant fibers have received the most attention because they are renewable and their properties including low density makes it possible to achieve high specific strength composite design [12,13,14,15,16,17]. 

Among the family of plant fibers considered for polymer reinforcement, pineapple leaf fiber (PALF) has superior mechanical properties and may be a potential substitute for synthetic fibers because of its economical, non-toxic and sustainable attributes [18]. Compared to carbon fiber, the tensile strength (0.4–1.6 GPa) and stiffness (6.5–82 GPa) of PALF are lower and can be considered appropriate for PS application considering the moderate strength to weight and stiffness to weight requirement for the application [12,19,20]. 

Herein, the authors seek to report the flexural properties of Methyl methacrylate (MMA) resin reinforced with alkali-treated PALF. The effects of fiber volume fraction and fiber orientation on the mechanical properties of the novel PALF/MMA composite material are reported in this work. This work uses the reported properties of the characterized PALF from the authors’ previous work to inform a novel PALF/MMA composite design [21]. With increasing incidence of cardiovascular risk factors such as diabetes, a corresponding rise in lower-limb amputations is noted, especially in rural communities [22,23]. The possibility of acquiring a good quality PS becomes far-fetched because of the high-cost carbon fiber in these rural settings. However, using PALF as an enabling technology replacement can be suitable because it is cheaper, easier and more accessible. 

## 2. Materials and Method

### 2.1. Materials

**Figure 1:**
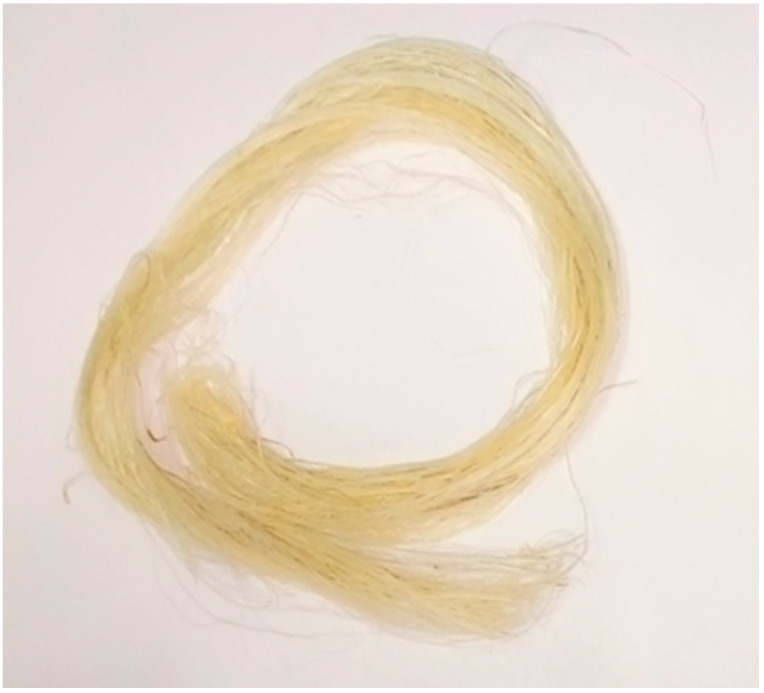
Extracted Pineapple leaf fiber (PALF).

The pineapple (*Ananas comosus*) fibers, Figure 1, were extracted from leaves supplied by Mawuli Farms, Nsawam, Ghana (5.8195° N, 0.3513° W). The mechanical properties of the characterized PALF are summarized in Table 1. The matrix composition used in this work was a mixture of Methyl methacrylate-based Prosthetic lamination resin (Orthocryl lamination resin) comprising 40–70% Methyl methacrylate (MMA) and 0.1–1% of N,N-Bis(2-hydroxypropyl)-p-toluidine serving as a hardener. Both constituents were purchased from Otto Bock Health Care, Salt Lake City, UT, USA. 

### 2.2. Methods

#### 2.2.1. Treatment of PALF

PALFs were isolated from pineapple leaves using a procedure that was described previously [21]. The isolated fibers were immersed in 6% wt/wt NaOH for 1 h. The fibers were then removed from solution, washed with deionized water and air-dried at 26 °C [24].

#### 2.2.2. Preparation of PALF/MMA Composites

Rectangular molds of dimension, 90 mm × 10 mm × 4 mm were designed using Plaster of Paris powder. The molds were greased and filled with varying amounts of fibers to make: 5%, 10%, 15%, 20%, 30%, 40% and 50% fiber volume fractions with fibers laid parallel to the length of the mold. This arrangement corresponds to 0° fiber orientation. The matrix component, MMA, and the hardener, N,N-Bis(2-hydroxypropyl)-p-toluidine, were premixed in a beaker in the ratio 100:6 wt/wt, respectively. The mixture was stirred for 2 min using a wooden spatula in a clockwise direction and left to settle for an additional 3 min to enable air bubbles trapped within the mixture to escape in order to achieve a bubble-free mixture. The homogenous mixture of resin was poured into the molds. Three samples were prepared for each fiber volume fraction. The experiment was repeated with fibers oriented at 45° and 90° to the length of the mould. The PALF/MMA mixture was left to cure for 24 h at 26 °C.

#### 2.2.3. Flexural Test

Three-point flexural tests were conducted on 0%, 5%, 10%, 15%, 20%, 30%, 40% and 50% *v*/*v* PALF/MMA composites having 0, 45 and 90° fiber orientations according to ASTM D790 using Mark-10 ESM301 Force Test Stand by Mark-10, Copiague, New York, USA, in Basic mode equipped with 1.5 kN load capacity and flexural testing fixtures [25]. All samples were conditioned at 26 °C and at relative humidity of 76% for 48 h before testing. With a support span length of 64 mm, the test was performed at a constant crosshead displacement rate of 10 mm/min at a relative humidity of 76% and at a temperature of 26 °C. For each composite formulation, three samples were tested. Determination of flexural properties used force-deflection data acquired directly from MESUR Lite software by Mark-10, Copiague, NY, USA. The deflection of the bar with respect to strain was calculated using Equation (1):

(1) $$D\;=\;\frac{rL^{2}}{6d}\;$$

where *D* (mm) is the deflection of the rectangular beam, *r* is the strain of the outer surface of the rectangular beam, *L* (mm) is the length of the support span and *d* (mm) is the depth of the rectangular beam. The flexural strength for each of the samples tested was calculated using Equation (2):

(2) $$\sigma_{f}\;=\frac{3PL}{2bd^{2}}$$

where *σ_f_* (MPa) is the stress in the outer surface of the beam, *P* (N) is the load at a given point on the load-deflection curve, *L* (mm) is the length of the support span, *b* (mm) is the width of the beam and *d* (mm) is the thickness of the beam tested.

#### 2.2.4. Micromechanical Models 

##### Rule of Mixture (RoM)

Voigt described a rule for predicting the elastic modulus of a composite using the elastic moduli and volume fractions of the reinforcement material and the matrix in the composite [9,26,27]. The rule of mixtures (RoM) assumes no void condition of the formed composite and that the constituent materials (reinforcement and matrix) are perfectly bonded together as a unit. Mathematically, the RoM is presented as Equation (3):

(3) $$E_{c}=E_{f}V_{f}+E_{m}\left(1-V_{f}\right)$$

where *E_c_, E_f_* and *E_m_* represent the Young’s modulus of the composite, fiber and matrix, respectively. 

##### Modified Rule of Mixtures (MRoM)

The MRoM is a modification of the RoM which takes into account the angle of orientation, *θ*°, of the fiber to the direction of stress [28]. Krenchel introduced a fiber orientation factor, *η_o_*_,_ which accounts for the contribution of the fiber to the stiffness of the composite with respect to the stress direction. Mathematically, the MRoM is represented as Equations (4) and (5):

(4) $$E_{c}=\eta_{o}E_{f}V_{f}+E_{m}\left(1-V_{f}\right)$$

(5) $$\eta_{o}=\underset{n}{\sum }a_{n}Cos^{4}\left(\theta_{n}\right)$$

where *E_c_, E_f_* and *E_m,_* represent the Young’s modulus of the composite, fiber and matrix, respectively. In Equation (5), *η_o_, α_n_* and *θ* represent the fiber orientation factor, the volume fraction of fiber oriented at θ to the direction of stress. 

##### Halpin–Tsai (H–T) Model

The Halpin–Tsai model is a semi-empirical model developed by curve fitting of experimental and elasticity-based model data [9,26]. The Halpin–Tsai equation incorporates the parameter, *ξ*, called the reinforcing factor, which depends on fiber geometry, fiber packing geometry and the loading condition. To estimate the modulus of the composite, Equations (6) and (7) are applied.

(6) $$E_{c}=\left[\frac{1+\xi \eta V_{f}}{1-\eta V_{f}}\right]E_{m}$$

(7) $$\eta =\frac{(E_{f}/E_{m})-1}{\left(E_{f}/E_{m}\right)+\xi }$$

where *E_c_, E_f_ and E_m,_* represent the Young’s modulus of the composite, fiber and matrix, respectively. *ξ* and *η* denote the reinforcing factor and ratio of the relevant fiber and matrix moduli and of the reinforcement factor.

#### 2.2.5. Statistical Data Analysis

The data acquired was organized and analyzed using IBM SPSS Statistical Software Version 22 (SPSS Inc., Chicago, IL, USA) and Origin 9 Data Analysis and Graphing Software (OriginLab Corp., Northampton, MA, USA). The distribution of the data was evaluated for normality assumption using Q-Q plots, Kolmogorov–Smirnov (KS) and Shapiro–Wilk tests at *p* > 0.05 before parametric analysis was conducted. Mean and standard deviations were used to describe the data. One-way independent analysis of variance (ANOVA) with Tukey’s HSD post hoc analysis was conducted to measure the effect of fiber volume fraction on the flexural properties of the composites at a 0.05 significance level.

## 3. Results

### 3.1. Flexural Test

**Figure 2:**
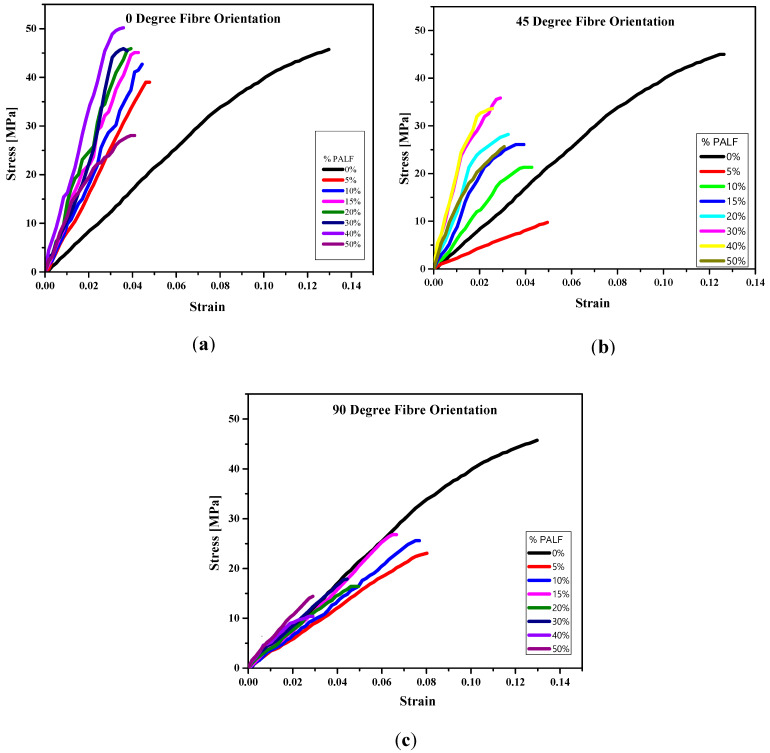
Stress–strain curves for PALF/MMA composites with varying fiber volume fractions oriented at: (**a**) 0°, (**b**) 45° and (**c**) 90°.

Figure 2a–c presents the stress and strain curves obtained from the flexural test performed. It was realized that the stress–strain curves obtained for all composites consist of an initial linear region (with the slope indicating the stiffness of the sample) and a non-linear portion at the peak where composite failure occurred. The flexural behavior in Figure 2a–c shows that all PALF/MMA composites had their flexural behaviour altered to varying degrees depending on fiber volume fraction and fiber orientation. In Figure 2a, at 0° fiber orientation, all the PALF composites exhibited a greater stiffness compared to the neat matrix (black curve). Figure 2b indicates that at 45° fiber orientation, the PALF composites also exhibited a higher stiffness than the neat matrix except for the 5% wt/wt composite (red curve). Figure 2c shows that at 90° fiber orientation, all the composites exhibited lower stiffness than the neat matrix, except for composites with higher fiber volume fraction (30–50% wt/wt). While stiffness generally increased, especially for the 0 and 45° fiber-oriented composites, the composite strengths were compromised, especially for the 45 and 90° fiber-oriented composites. This indicates that the fiber orientation affected the composite flexural strength and stiffness.

In Table 2, the results of increasing PALF volume fraction and fiber orientation on composite strength (CS), composite flexural modulus (CFM) and strain at failure are presented. A CS of 45 MPa was recorded for the neat matrix. For lower PALF percent volume composites, 5–15% *v*/*v*, the results showed a similar pattern of decreasing CS compared to the neat matrix for all fiber orientations. For 20–50% *v*/*v* composites, the strength recorded showed varied patterns depending on fiber orientation. For the 0° fiber-oriented composites, the CS were barely altered for 20 and 30% *v*/*v* composites. However, it increased to 51 MPa for the 40% *v*/*v* composite which was 13% higher than the strength of the neat matrix. A significant (*p* < 0.05) decrease in CS to 28 MPa was recorded for the 50% *v*/*v* composite, representing 40% decrease in CS relative to the neat matrix. For the 45° fiber-oriented composites, the maximum CS recorded was 36 MPa for 30% *v*/*v* composite, however, this was 20% lower than the neat matrix. The CS further decreased to 34 and 26 MPa for the 40 and 50% *v*/*v* composites, respectively. This implied that none of the 45°–PALF composites achieved a better CS than the neat matrix. The CS of 20–50% *v*/*v* composites for the 90° fiber-oriented composites were erratic with the lowest CS (10 MPa) recorded for the 40% *v*/*v* composite. For each type of fiber-oriented composite, the changes in CS are correlated to the fiber volume fraction, however, among the three types of fiber-oriented composites, the CS are correlated to the fiber orientation.

The effect of PALF volume fraction on CFM is presented in Table 2. The flexural modulus recorded for the neat matrix was 429 ± 20 MPa. A steady increase in CFM from 740 ± 80 to 1692 ± 186 MPa was recorded for the 0° fiber-oriented composite. This represented 75 and 294% improvement in the flexural modulus of the neat matrix for the 5% and 40% PALF composites, respectively. For the 45° fiber-oriented composites, as shown in Table 2, an increase in CFM was recorded from 199 ± 12 to 1677 ± 101 MPa with the minimum and maximum CFM corresponding to 5% and 30–40% PALF *v*/*v* composites, respectively. The CFM for 30 and 40% PALF composites were barely altered. Whereas all 45° fiber-oriented composites recorded CFMs higher than the neat matrix, only the 5% PALF *v*/*v* composite recorded a lower CFM than the neat matrix. For the 90° fiber-oriented composites, the minimum and maximum CFM recorded were 308 ± 34 and 565 ± 62 MPa, respectively. The minimum CFM was recorded for the 5% PALF *v*/*v* composite whereas the maximum CFM was recorded for the 40% PALF *v*/*v* composite. 

The results on percentage strain at failure presented in Table 2 indicated a similar decreasing pattern of strain at failure for the three types of fiber orientations. For each fiber orientation, a significant (*p* < 0.05) decrease in percentage strain at failure for all composites relative to the neat matrix was recorded. Thus, the incorporation of the fiber into the matrix decreased the strain at failure of the matrix, an indication of lower ductility. While the percentage strain at failure ranged from 3–5% and 2–5% for 0° and 45° fiber-oriented composites, respectively, that for the 90° fiber-oriented composites ranged from 2–8%. Even though the percentage strain at failure seems to be similar for composites with fiber orientations of 0 and 45°, as shown in Table 2, the strength and stiffness exhibited by the composites with 0° fiber orientation are preferred because they are higher than those recorded for 45 and 90° fiber-oriented composites.

### 3.2. Micromechanical Model

**Figure 3:**
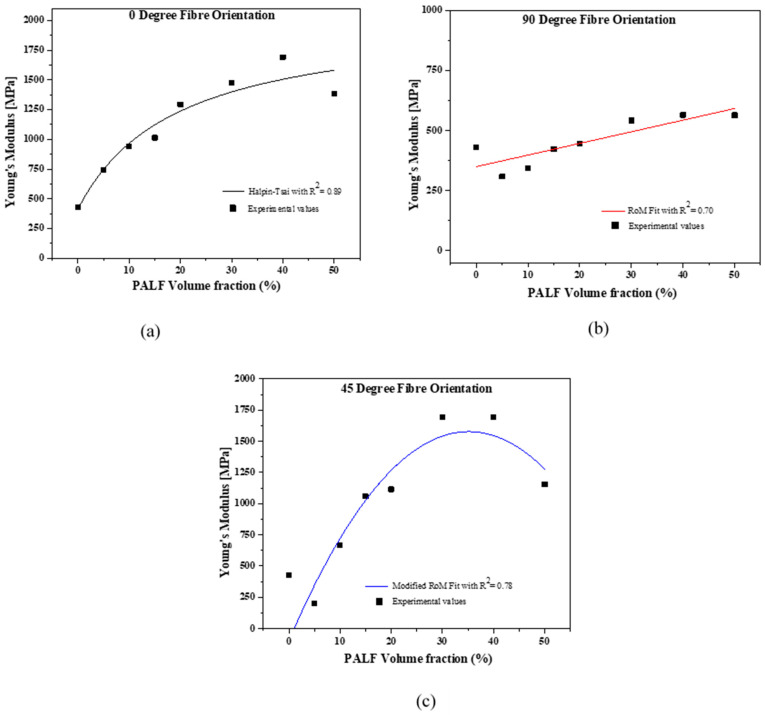
Experimental Young’s moduli of cured PALF/MMA composites fitted by (**a**) Halpin–Tsai, (**b**) Modified Rule of Mixture and (**c**) Rule of mixture as a function of fiber volume fraction oriented at 0, 45 and 90°, respectively.

The experimental data of the flexural modulus for the three fiber-oriented composites were fitted to three micromechanical models to determine the level of correlation. It was realized that each model fitted differently for each fiber-oriented composite. The best fit for 0° fiber-oriented composite was the Halpin–Tsai (H-T) model, as shown in Figure 3a, with an extracted R^2^ value of 0.89. Figure 3b indicates the rule of mixtures (RoM) to be the best fit model for the 90° fiber orientated composites with an R^2^ value of 0.70. For 45° fiber-oriented composites, as shown in Figure 3c, indicated the modified rule of mixtures (MRoM) as the best fit model with R^2^ value of 0.78. Unlike the H-T and MRoM that showed an initial linear behaviour at lower fiber volume fractions and non-linear behaviour at higher fiber volume fractions, the RoM indicated a linear relationship between composite stiffness and fiber volume fraction.

### 3.3. Determining the Minimum, Critical and Maximum Fiber Volume for PALF/MMA Composites

According to composite theory, the overall mechanical properties of a composite are influenced by the mechanical properties of the fiber and matrix elements [26,27,29]. It is realized that, if there are very few fibers (ν*_f.min_*) within the matrix, the stress on the composite may be high enough to break the fibers [9]. Thus, the non-load bearing fibers can be regarded as an array of aligned holes which become stress concentration zones within the matrix. The result is that the composite strength and stiffness fall below those of the matrix. Therefore, there is a critical fiber volume fraction, (*ν_f.crit_*), threshold below which the fiber compromises the strength and stiffness of the matrix [9,26]. 

In the design of load-bearing structures using fiber-reinforced composites, it is imperative to determine the practical minimum (ν*_f.min_*), critical (*ν_f.crit_*) and maximum (*ν_f.max_*) fiber volume fractions. For application, it is necessary to design for ν*_f_* > ν*_f.crit_* [9] where strength and stiffness are the desired properties. Table 3 shows the practical ν*_f.min_*_,_ ν*_f.crit_* and *ν_f.max_* of PALF/MMA composites for the three different fiber orientations. The ν*_f.min_*, ν*_f.crit_* and ν*_f.max_* values were extracted from the composite strength results in Table 2.

## 4. Discussion

### 4.1. Effect of PALF Fiber Orientation and Volume Fraction on Flexural Properties of PALF/MMA Composite

Plant fibers are known to improve the mechanical properties of polymeric materials [27]. The improvement in mechanical properties of the polymeric matrix is possible because of the superior mechanical properties of these plant fibers [27,30]. Plant fiber thermoset composites form a brittle-fiber ductile-matrix system where the composite strength and stiffness are determined by the fiber volume fraction and fiber orientation [9,31]. The fundamental composite theory, RoM, suggests that increasing the fiber volume fraction increases the composite strength and stiffness [32]. However, there is a practical maximum (ν*_f.max_*) fiber volume above which composite properties deteriorate due to either porosity, poor interfacial bond between fiber and matrix or fiber-fiber interaction. Loads are best transmitted along the fiber when the fiber is aligned in the direction of the applied stress. Fibers oriented away from the direction of stress become stress concentration zones since their orientation do not encourage smooth stress transfer from matrix to fiber [9].

In this work, composite strength (CS) and composite flexural modulus (CFM) generally increased with increasing fiber volume fraction to a limit for the three types of fiber orientations. However, a decrease in CS was observed for the three types of fiber-oriented composites with 5% *v*/*v* PALF. For CFM, only composites with fiber orientations at 45° and 90° recorded a decrease in CFM for 5% *v*/*v* PALF/MMA composite. The decrease in CS and CFM at lower fiber volume fraction occurred because within the matrix, the fibers (5% *v*/*v*) are believed to have behaved as a series of aligned holes which acted as stress concentration zones and decreased the CS by 15, 78 and 46% for composites with fiber orientations of 0°, 45° and 90°, respectively [9]. The observed decrease in CS is in good agreement with composite theory [9] and has been reported in another research [28]. Further increasing the fiber volume fraction (ν*_f_* > 5%) increased the CS and CFM with the highest CS (50 MPa) recorded for the 40% *v*/*v* 0° fiber-oriented composite, representing 13% improvement in the CS of the neat matrix. While an increment in CS was observed for composites with 45° and 90° fiber orientations, none of the composites achieved a better CS compared to the neat matrix. The inability of the composites with fiber orientations of 45 and 90° to improve the CS of the matrix was because the fiber orientations did not encourage stress transfer and transmission. Instead, the fibers behaved as an impedance to stress transfer and transmission, thus creating stress concentration zones within the composite which decreased the CS.

All the 0° fiber-oriented composites achieved higher CFM than the neat matrix with the highest CFM recorded for the 40% *v*/*v* composite (1687 MPa), representing 294% improvement in CFM. The 45° fiber-oriented composites also recorded higher CFM for all composites except for the 5% *v*/*v* composite. The decrease in CFM for 5% *v*/*v* can again be linked to the generation of stress concentration zones due to the low fiber volume fraction. However, a 290% improvement in CFM was realized for both 30 and 40% *v*/*v*, 45° fiber-oriented composites. The result suggests that CS and CFM is correlated with fiber volume fraction. However, beyond 40% *v*/*v* PALF composition, the CFM decreased for all three types of composites. The decrease can be attributed to the fiber-fiber interaction occurring at higher fiber volume fractions, similar to results from the work of Asumani et al. [33]. A decreasing trend in percentage failure strain is recorded for the three types of orientations that is in good agreement with the inverse relationship between stiffness and strain at failure as postulated by composite micromechanics [27,33].

Given the low range (0.6–1.2 kN) of weight exerted on the PS and comparing the strength to weight (1.4–2.9 GPa/g/cm^−3^) and the stiffness to weight (102.6–105.3 GPa/g/cm^−3^) of carbon fiber to that of PALF; (0.3–1.1 GPa/g/cm^−3^) and (4.3–53.6 GPa/g/cm^−3^), it may be appreciated that PALF would be an appropriate reinforcing material given the low cost and low energy required for its production. Further to this point, the flexural test results suggest that, the novel PALF/MMA composite can be used for a variety of biomedical structural applications such as PS development. Additionally, results on the effect of fiber orientation showed 0° fiber orientation to be the best orientation to yield the optimum CS and CFM for the composites.

### 4.2. Correlation between the Micromechanical Models and the Experimental Results

The experimental values of the three composites fitted differently in all the models tested in this study; namely, Rule of mixtures (RoM), Modified rule of mixtures (MRoM) and Halpin–Tsai (H-T). The H-T model is a semi-empirical model that incorporates the physical properties of the fiber; namely, fiber geometry, packing geometry and loading condition into predicting the composite’s modulus [26,34]. The effect of the physical composite properties is captured by the efficiency factor, *ξ*, which in this work was taken to be 2. An efficiency factor of 2 was used in the model having considered PALF to have a circular cross section and a square array packing order. The H-T model best predicted the 0° composites at lower fiber volumes (5–10% PALF *v*/*v*) which is in good agreement with literature [32]. The MRoM model applied herein considered the fiber orientation distribution factor, η_o_, according to Krenchel theory [32,34,35]. While the H-T model showed that stiffness would likely plateau at higher fiber volumes, the MRoM showed that, at higher fiber volume fractions (v*_f_* ≥ 50% *v*/*v*), the CFM would most likely decrease. The decrement at higher fiber volume fraction was observed in the experimental results for the 50% *v*/*v* 45° fiber-oriented composite which can be linked to the fiber-fiber interaction effect associated with higher fiber volume fractions. The RoM showed a linear relation between v*_f_* and CFM. Against the experimental values, the RoM seemed to better predict the CFM for the 15–40% *v*/*v* PALF/MMA 90° fiber-oriented composites. It is noted that all the three models are based on perfect fiber arrangement, packing geometry, fiber-matrix bonding and loading conditions. The challenges in having these perfect conditions in practice may have affected the level of correlations (R^2^ values) obtained for each fitting.

### 4.3. Maximum Practical Fiber Volume (ν_f.max_) Relevant for PALF/MMA Composite Application

Based on composite mechanics, there is a practical maximum fiber volume fraction responsible for the optimum strength and modulus of the composite [9]. In this work, the determined ν*_f.max_* for 0°, 45° and 90° fiber-oriented composites were varied based on fiber orientations. It was realized that the farther the fiber is oriented away from the stress, the greater the effect of stress concentration zones which occur at lower fiber volume fractions. This effect resulted in the failure of the composites at lower fiber volume fractions as observed for the 45° and 90° fiber-oriented composites [35]. However, for the 0° fiber-oriented composites, the fibers were aligned in the direction of the stress, hence the optimum strength and modulus were obtained at a higher fiber volume fraction (40% *v*/*v*). Unlike the 45° and 90° fiber-oriented composites where failure resulted from stress concentration zones, the failure for the 0° fiber-oriented composites can be attributed to fiber-fiber interaction resulting from the saturation of the fiber-matrix system. Compared to another work, the maximum fiber volume fractions determined for flax and jute polyester composites were 33 and 47%, respectively [28].

## 5. Conclusions

In this work, the flexural properties of PALF/MMA composites were studied, presenting an opportunity for low-cost, facile production of prosthesis sockets. The optimum composite strength and stiffness were determined for potential prosthetic socket application based on fiber volume fraction and fiber orientation. The results showed that in financially constrained regions, locally available and low-cost PALF can be a good alternative for PS application where the stiffest material is not necessary, but a material that is stiffer in some areas and softer to accommodate the body weight is required. Additionally, in areas where polymer access is an issue, a composite system made with significantly less MMA, but having a majority of the desired mechanical properties, can be obtained. Results from the micromechanical models indicated the experimental data to have fitted differently based on composite fiber orientation. Based on the range of weight exerted on a prosthetic socket, the moderate strength to weight and stiffness to weight property, and the low cost of PALF, it is expected that with 0° fiber orientation and a 40% fiber volume fraction, a good quality low-cost prosthetic socket can be fabricated for amputees, especially in resource-constrained parts of the world.

## Figures and Tables

**Table 1:** Mechanical Properties and Alkali Treatment Conditions of the PALF used as reinforcement in this work [21].

Mechanical Property	PALF
Density (g/cm^3^)	1.53
Diameter (μm)	61 ± 0.025
Tensile strength (MPa)	1620 ± 150
Young’s modulus (GPa)	27 ± 0.9
Strain at break (%)	6.7 ± 0.5
Concentration of alkali treatment of PALF (*w*/*w*)	6%
Treatment time (h)	1

**Table 2:** Mean flexural strength, modulus and strain at failure of PALF/MMA composite with 0, 5, 10, 15, 20, 30, 40 and 50 *v*/*v* PALF at 0, 45 and 90° fiber orientation. Values are the means of three experiments with a standard error.

Fiber Volume Fractions (% *v*/*v*)	Flexural Strength (MPa)			Flexural Modulus (MPa)			Percentage Strain at Failure (%)		
	Mean ± SD			Mean ± SD			Mean ± SD		
	Fiber Orientation (°)			Fiber Orientation (°)			Fiber Orientation (°)		
	0	45	90	0	45	90	0	45	90
0	45.0 ± 3.6 ^a^	45.0 ± 3.6 ^a^	45.0 ± 3.6 ^a^	429.0 ± 34.3 ^a^	429.0 ± 34.3 ^a^	429.0 ± 34.3 ^a^	13 ± 1.3 ^a^	13 ± 1.3 ^a^	13 ± 1.3 ^a^
5	39.0 ± 2.4 ^b^	9.8 ± 1.1 ^b^	23.6 ± 1.5 ^b^	741.8 ± 81 ^b^	199.2 ± 12.0 ^b^	308.4 ± 33.9 ^b^	5.0 ± 0.5 ^c^	5.0 ± 0.2 ^b^	8.0 ± 0.9 ^b^
10	42.7 ± 6.0 ^a^	21.3 ± 2.3 ^c^	25.6 ± 0.2 ^b^	940.1 ± 103 ^c^	666.8 ± 40 ^c^	343.4 ± 37.8 ^c^	4.5 ± 0.9 ^c^	4.3 ± 0.9 ^c^	7.5 ± 0.3 ^b^
15	45.1± 10.1 ^a^	26.1 ± 5.1 ^d^	26.8 ± 1.8 ^c^	1013.8 ± 111 ^d^	1058.0 ± 63 ^d^	422.4 ± 46.5 ^d^	4.1 ± 0.8 ^c^	3.8 ± 0.8 ^c^	6.5 ± 0.6 ^c^
20	45.6 ± 7.2 ^a^	28.2 ± 2.5 ^d^	16.4 ± 0.5 ^d^	1294.3 ± 142 ^e^	1114.4 ± 67 ^e^	445.0 ± 49.0 ^e^	3.9 ± 0.7 ^c^	3.3 ± 0.3 ^c^	4.9 ± 0.1 ^d^
30	45.5 ± 5.3 ^a^	35.9 ± 3.2 ^e^	17.9 ± 3.8 ^d^	1473.5 ± 162 ^f^	1677.6 ± 186 ^f^	542.3 ± 59.7 ^f^	3.6 ± 0.9 ^c^	2.9 ± 0.9 ^d^	4.4 ± 0.1 ^d^
40	50.2 ± 4.9 ^a^	33.6 ± 2.5 ^e^	10.4 ± 1.1 ^e^	1692.0 ± 186 ^g^	1677.6 ± 101 ^f^	565.0 ± 62.1 ^g^	3.3 ± 0.2 ^d^	2.6 ± 0.6 ^d^	2.9 ± 0.4 ^e^
50	28.1 ± 2.5 ^c^	25.7 ± 4.0 ^d^	14.4 ± 3.0 ^d^	1382.4 ± 152 ^h^	1155.5 ± 69 ^f^	563.9 ± 62.0 ^g^	4.0 ± 0.5 ^c^	3.1 ± 0.3 ^d^	2.8 ± 0.6 ^e^

The a, b, c, d, e, f, g and h indicates a significance difference between samples (fiber volume fractions) in each group (orientation) at the 0.05 confidence level. Flexural strength, modulus and percentage strain at failure are represented using mean and standard deviation (±SD).

**Table 3:** Summary of practical ν*_f.min_*, ν*_f.crit_* and ν*_f.max_* of PALF/MMA composites for fiber orientations of 0°, 45° and 90°.

Fiber Orientation (°)	ν*_f.min_* (%)	ν*_f.crit_* (%)	ν*_f.max_* (%)
0	5	20	40
45	5	Not realized	30
90	5	Not realized	15

Not realized: All the CS recorded are below the flexural strength of the neat matrix.

## Data Availability

All data are in the paper.

## Abbreviations

**Acronym**	**Meaning**
PALF	Pineapple leaf fiber
PS	Prosthetic Socket
MMA	Methyl methacrylate
FRC	Fiber Reinforced Composite
CS	Composite Strength
CFM	Composite flexural modulus
RoM	Rule of mixture
MRoM	Modified Rule of mixture
H-T	Halpin–Tsai
*ν._min_*	Minimum fiber volume
*ν._max_*	Maximum fiber volume
*ν._crit_*	Critical fiber volume
